# Data set for Gambung green tea aroma using on electronic nose

**DOI:** 10.1186/s13104-024-06905-6

**Published:** 2024-09-03

**Authors:** Dedy Rahman Wijaya, Rini Handayani, Muhammad Dzakyyuddin Badri, Shabri Shabri, Vitria Puspitasari Rahadi

**Affiliations:** 1 Present Address: School of Applied Science, Telkom University, Bandung, West Java Indonesia; 2 Present Address: Research Institute for Tea and Cinchona, Gambung, West Java Indonesia

**Keywords:** Electronic nose, Gas sensor, Green tea quality, Machine learning

## Abstract

**Objectives:**

In recent years, there has been much discussion and research on electronic nose (e-nose). This topic has developed mainly in the medical and food fields. Typically, e-nose is combined with machine learning algorithms to predict or detect multiple sensory classes in each tea sample. Therefore, in e-nose systems, e-nose signal processing is an important part. In many situations, a comprehensive set of experiments is required to ensure the prediction model can be generalized well. This data set specifically focuses on two main goals such as classification of green tea quality and prediction of organoleptic score. In this experiment, Gambung dry green tea samples were used. The challenge is that dry tea does not emit as strong an aroma as tea infusions, making it more difficult for the e-nose system to detect and identify the aromas. This data set offers a valuable resource for researchers and developers to conduct investigations and experiments by classifying and detecting organoleptic scores that aim to categorize and identify organoleptic ratings. This enables a deeper understanding of the quality of dry green tea and encourages further integration of e-nose technology in the tea industry.

**Data description:**

This experiment focused on analyzing green tea aroma using six gas sensors. Seventy-eight green tea samples were tested, each observed three times, using a tea chamber connected to a sensor chamber via a hose and an intake micro air pump. Air flowed from the tea chamber to the sensor chamber for 60 s, followed by 60 s of aroma data recording. This data was saved into CSV files and labeled according to the Indonesian National Standard (SNI) 3945:2016, which includes special and general requirements for green tea quality. An organoleptic test by a tea tester further labeled the data set into “good” or “quality defect” for classification and provided organoleptic scores based on dry appearance, brew color, taste, aroma, and dregs of brewing for continuous label.

## Objective

By detecting and classifying organoleptic scores that seek to identify and categorize organoleptic ratings, this data set is helpful material for researchers and developers to carry out studies and experiments. This facilitates a deeper understanding of the quality of dry green tea and promotes e-nose technology integration in the tea industry. Researchers are increasingly utilizing e-nose to automatically evaluate tea quality. These devices detect various aromas using sensors and analyze the unique scents of different substances. By distinguishing different types of tea based on their aromas, e-noses provide a promising method for objectively and automatically assessing tea quality and organoleptic scores [1]. Using the dry method to test the quality of green tea has the advantage of being more practical and does not require complicated procedures like the steeping method. It does not require special attention to the water source, temperature, soaking duration, or the process of separating the water and tea grounds, reducing the possibility of human error in testing [2].

## Data description

Green tea samples were obtained from the Gambung Tea Plantation located in Ciwidey, Bandung Regency, West Java. At the foot of Mount Tilu, there is the Tea and Cinchona Research Institute (PPTK). The Gambung Tea Plantation has a land area of around 600 hectares, most of which is the Gambung tea plantation, and the rest is natural forest. This plantation is a production area for Assamica tea and Gambung Sinensis series tea [3]. The coordinates of the tea plantation are − 7.143291440042576, 107.51636224602858.

The values in this data set represent the aroma of each Gambung green tea sample in pekoe dry preparation. The aroma is sensed by six gas sensors made of metal-oxide semiconductors (MOS) that are externally supplied by 3.3VDC. Based on the sensing of tea testers summarized in the organoleptic score, there are two classes, namely “good” and “quality defects”. The file “*.xlsx” contains the data set that has been sampled. There is several columns in the data set as follows [4]:

- Sampling_id: describes chop/sample id.
- MQ_3: response of MQ3 gas sensor.
- MQ_5: response of MQ5 gas sensor.
- MQ138: response of MQ138 gas sensor.
- TGS822: response of TGS822 gas sensor.
- TGS2602: response of TGS2602 gas sensor.
- TGS2620: response of TGS2620 gas sensor.
- Score: organoleptic score for continuous label.
- Class: green tea quality (“good” or “defect”) for discrete label.

The process of recording the response of those gas sensors according to green tea samples is controlled by the ATMEGA328 microcontroller. The microcontroller features used in this research are the analog signal reading feature from the gas sensors through the ADCs (Analog to Digital Converters) and asynchronous serial communication to transmit the data read from the ADCs to the computer for further processing. The ADC has a resolution of 10 bits, where an analog input voltage is transformed into a 10-bit digital representation using the method of successive approximation. This resolution indicates that the voltage level will be separated into 2^10^ or 1024 different levels. Therefore, the reading value to begin somewhere within the range of 0 to 1023. This reading value is presented by the microcontroller using the variables that it has access to. The value of the voltage level can be obtained by doing the following:

1 $$\begin{array}{l}\:Vsample=\\\:\frac{Sensor\:Reading\:Value}{1024}\:x\:3.3\:VDC\end{array}$$

In this experiment, a total of 78 different tea samples were subjected to testing, with each tea sample undergoing three separate observations. The apparatus employed consists of two key chambers such as a sample chamber and a sensing chamber. The sample chamber is equipped with a single opening connected to a silicone hose, allowing for the passage of air into the sensing chamber. In contrast, the sensing chamber features three openings for different purposes: first for connecting to the sensor array wiring and control system, second for the air inlet from the sample chamber, and third for expelling air back into the open air. These access points in both chambers have been meticulously designed to minimize any potential air leakage.

For each data collection instance, 15 g of Gambung green tea samples are placed within the sample chamber, which is constructed from borosilicate glass. Utilizing a 12VDC air pump, air from the sample chamber is drawn into the sensing chamber over a 60-second duration. Subsequently, within the sensing chamber, this sampled air is examined by six gas sensors for another 60 s, and the resulting data is stored in CSV format. Each tea sample was observed three times. After that, all experimental data were summarized into an MS Excel Spreadsheet (xlsx) to make processing easier. Each of these samples is treated as an independent data point, and this procedure is performed to ensure that data is captured after inhalation and before exhalation processes. This meticulous approach is essential to introduce greater variance into the data for training machine learning models and prevent overfitting. Following the sampling process’s completion, the sampled air is expelled through an exhaust hose into the open air for 60 s, maintaining a neutral environment within the sensing chamber. This file is subsequently processed and labelled for training purposes. Table 1 shows the overview of data sets.

**Table 1:** Brief description of data set

Label	Name of data set	File types (file extension)	Data repository and identifier (DOI or accession number)
Data file 1	gambung_green_tea_78_chops	MS Excel file (.xlsx)	Harvard Dataverse (10.7910/DVN/BGIVM8) [4]
Data file 2	Component list	MS Excel file (.xlsx)	Harvard Dataverse (10.7910/DVN/BGIVM8)[4]
Data file 3	e-nose schema	Image (.png)	Harvard Dataverse (10.7910/DVN/BGIVM8)[4]

## Limitations

The data set is gathered under controlled temperature conditions, but there is no control over humidity.

## Data Availability

The data described in this Data note is openly accessible on Harvard Dataverse at the following URL: 10.7910/DVN/BGIVM8. For more comprehensive information and direct data access links, please consult Table 1 and refer to the citations in reference [4].

## Abbreviations

e-nose: Electronic nose
MOS: Metal-oxide semiconductor
VDC: Volt Direct Current
